# Long-Term Fatigue and Its Probability of Failure Applied to Dental Implants

**DOI:** 10.1155/2016/8927156

**Published:** 2016-07-19

**Authors:** María Prados-Privado, Juan Carlos Prados-Frutos, Sérgio Alexandre Gehrke, Mariano Sánchez Siles, José Luis Calvo Guirado, José Antonio Bea

**Affiliations:** ^1^Department of Medicine and Surgery (Stomatology Area), Rey Juan Carlos University, C/ Tulipán s/n, Móstoles, 28933 Madrid, Spain; ^2^Applied Modelling and Instrumentation Group, Aragón Institute of Engineering Research, University of Zaragoza, C/ Mariano Esquillor s/n, 50018 Zaragoza, Spain; ^3^Biotecnos Research Center, Rua Dr. Bozano 571, 97015-001 Santa Maria, RS, Brazil; ^4^University Catholic San Antonio de Murcia (UCAM), Guadalupe, 30107 Murcia, Spain; ^5^Oral Medicine, University of Murcia, 30001 Murcia, Spain; ^6^International Dental Research Cathedra, Faculty of Medicine and Dentistry, University Catholic San Antonio de Murcia (UCAM), Guadalupe, 30107 Murcia, Spain

## Abstract

It is well known that dental implants have a high success rate but even so, there are a lot of factors that can cause dental implants failure. Fatigue is very sensitive to many variables involved in this phenomenon. This paper takes a close look at fatigue analysis and explains a new method to study fatigue from a probabilistic point of view, based on a cumulative damage model and probabilistic finite elements, with the goal of obtaining the expected life and the probability of failure. Two different dental implants were analysed. The model simulated a load of 178 N applied with an angle of 0°, 15°, and 20° and a force of 489 N with the same angles. Von Mises stress distribution was evaluated and once the methodology proposed here was used, the statistic of the fatigue life and the probability cumulative function were obtained. This function allows us to relate each cycle life with its probability of failure. Cylindrical implant has a worst behaviour under the same loading force compared to the conical implant analysed here. Methodology employed in the present study provides very accuracy results because all possible uncertainties have been taken in mind from the beginning.

## 1. Introduction

Implants are widely used, as Misch discussed in [1], “*to restore the patient to normal contour, function comfort, esthetics, speech, and health, whether restoring a single tooth with caries or replacing several teeth. What makes implant dentistry unique is the ability to achieve this goal regardless of the atrophy, disease, or injury of the stomatognathic system.*”

The use of dental implants to replace missing teeth has become a routine in dental practice. Despite dental implants have a high success rate [2], there are a lot of factors that can involve complications and failure. On occasion, prosthetic implants fail because of mechanical and biological causes [3]. The primary causes of implant failure in clinical observations include incomplete osseointegration [4], infection, and impaired healing [5]. In addition to this, the failure of dental implants can be attributed to poor planning or the use of an improper implant for a given region of the maxilla or mandible [6, 7]. Occlusal conditions such as parafunctional habits have been identified as other important and potential causes of fracture.

Overload is, as it was said previously, an important factor in dental implant failure and one of the reason is bruxism. Misch explains in [1] that forces involved in bruxist person are significantly more important than normal physiologic masticatory loads. This situation affects above everything the teeth, bone, implants, and prostheses although its consequences depend on the bruxism type (diurnal or nocturnal). Although this parafunction increases the risk of failure in dental implants, bruxism does not necessarily represent a contraindication for implants, but it does dramatically influence treatment planning [1].

Nowadays, most of dental implants are made of titanium, both pure or alloy, which is a highly biocompatible biomaterial (both* in vitro* and* in vivo*) and show excellent performance balance between biofunctional, mechanical, and physicochemical properties [3, 8]. However, its rigidity as compared with alveolar bone is an important disadvantage of titanium. Due to the fact that it reduces the stresses in the bone, a loss of bone mass appears. An important implication of this bone loss is the risk of implant fracture [9].

Given that failure is an important occurrence in dental implants, several papers have been published where this problem has been treated from different points of view such as clinical studies and finite element analysis [10–14]. However, most of these studies have been done from a deterministic point of view. Due to the fact that fatigue in dental implants is very sensitive to many different parameters, a probabilistic fatigue analysis is crucial in order to have a more accurate prediction on probability of failure and mean life. The randomness of material properties and loads have been considered in this study, as well as its influence on the life of the structural components [15].

Fatigue phenomenon is known as the change that appears on materials when cyclic loads are applied. The International Organization for Standardization published in 1964 a report entitled General Principles for Fatigue Testing of Metals where fatigue was defined as “*a term which applies to changes in properties which can occur in a metallic material due to the repeated application of stresses or strains, although usually this term applies specially to those changes with lead to cracking or failure*” [16].

The fatigue life is the number of stress cycles required to cause failure. This number relies on several variables, such as stress level, stress state, cyclic wave form, fatigue environment, and metallurgical condition of the material [17]. Prediction of fatigue life can be difficult because small changes in the specimen or test conditions can significantly affect fatigue behaviour. Boyer detailed in [17] that fatigue cracking is normally the outcome of cyclic stresses. These stresses are sufficiently below the static yield strength of the material. Fatigue cracks initiate and propagate in regions where the strain is most severe. This area of high deformation becomes the initiation for a fatigue crack, which propagates under the applied stress through the material until the complete fracture.

**Figure 1:**
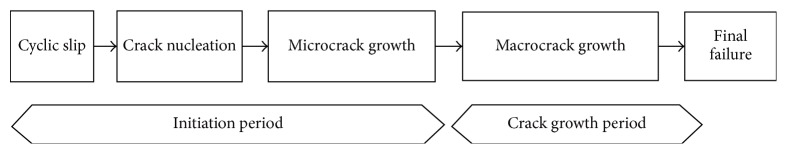
Different phases of fatigue life [18].

Fatigue process can be divided into two periods: the crack initiation period and the crack growth period, as Figure 1 shows. As Schijve detailed in [18] in the crack initiation period, fatigue is a material surface phenomenon and it finishes when microcrack growth is no longer depending on the material surface conditions [18].

In crack initiation testing, the specimen is exposed to the number of cycles required for a fatigue crack to initiate and to grow large enough to produce failure. In crack propagation testing, to determine the crack growth rates, fracture mechanics methods are used [17].

It is also known that fatigue life is more sensitive to this influence in the initiation period. In any case, laboratories try to eliminate these sources of variability in order to obtain more confident results, so fatigue tests are carried out under closely controlled conditions [18].

This paper shows a new method of studying long-term life in dental implants and its components both with normal conditions and functional overload. Authors propose here a new way of studying fatigue based on cumulative damage model and probabilistic finite elements. This method allows us to know what is the probability of failure in each cycle without doing any mechanical test as previously explained, or, what it is the same, the methodology employed here allows us to obtain the failure probability without breaking any implant.

## 2. Materials and Methods

The aim of the method explained in this section is to obtain the mean life and the probability of failure associated with each cycle without doing any fatigue test as previously explained. Novelty of this method is based, mainly, in the perspective from the study involved. Most of fatigue studies are addressed from a deterministic point of view, while we here consider the randomness of some variables, as load magnitude and direction or material properties (i.e., Young modulus). Once stress distribution under a particular condition is known, long-term life and probability of failure can be determined by employing a probabilistic model developed by Bogdanoff and Kozin and by the Stochastic Finite Elements Method [19–21]. To develop this method, the use of ANSYS® (version 14.5, Canonsburg, Pennsylvania, United States) and Mathematica® (version 10, Oxfordshire, United Kingdom) is only required.

Geometry in IGES format has been used to generate the finite element mesh employing the commercial software ANSYS® and once geometry was meshed, boundary conditions can be applied and stress analysis can be done. Use of finite element software makes the efforts to obtain stress distribution on dental implants under different load situations easier.

### 2.1. Dental Implants Characteristics

Dental implants employed in the present study are manufactured by Avenir S.L. (Rimini, Italy) and sold by Proclinic S.A. (Madrid, Spain), with the characteristics as described in Table 1. The implant name employed here is the same as the catalogue.

**Figure 2:**
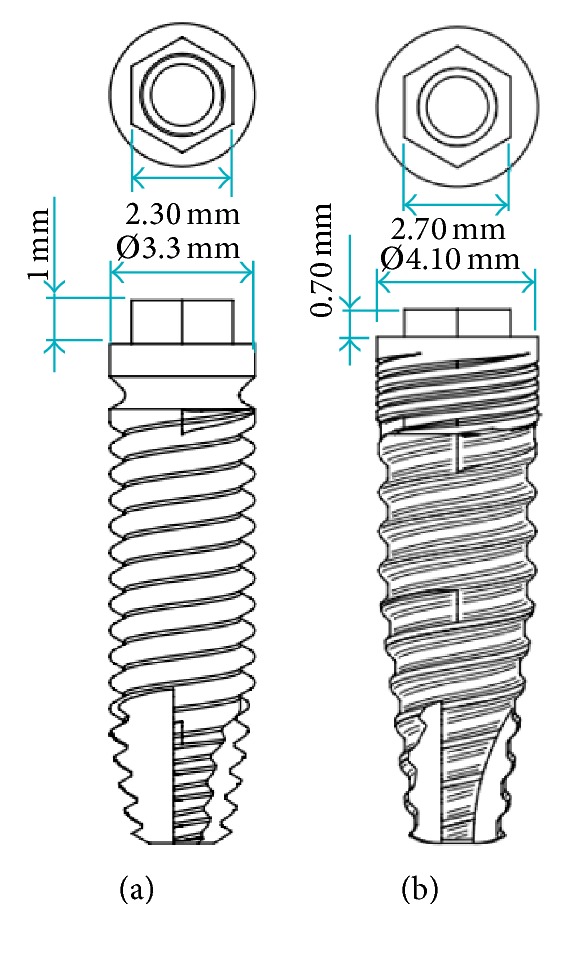
Dental implants characteristics: (a) IP861 and (b) IP804.

These two implants were used in this study: cylindrical external Ø3.30 mm (IP804) and conical external Ø4.10 mm (IP861).  Figure 2 illustrates the dimensions and appearance of the implants (14.5 mm in length).

### 2.2. Material Properties

Implants were modelled with linear, elastic, isotropic, and homogeneous properties. Both implants are made from Titanium Grade IV (Young modulus = 114 GPa, provided by the manufacturer).

### 2.3. Boundary Conditions and Loading Configuration

**Figure 3:**
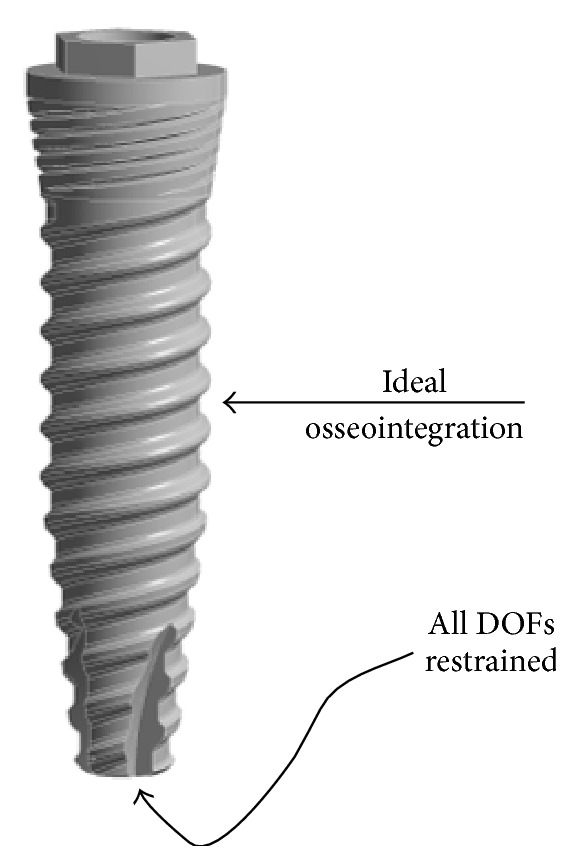
Boundary conditions applied in all situations.

All degrees-of-freedom (DOFs) were restrained in all directions at the nodes on the apical part of the implants and ideal osseointegration was simulated in the rest of the implant. Boundary conditions applied are shown in Figure 3.

Tables 2 and 3 detail bite force values in molar and anterior region found in the literature, where N represents the International System units for load. In the view of the literature, we have decided to employ in this study forces and angle detailed in Table 4.

Thus, the literature suggests maximum bite forces in the region area in the range of 300–629 and 65 N for adults and maximum forces in the anterior region between 146 and 178 N. In the current study, forces in two different regions with different angles were simulated: a static load of 489 N in the molar region and a load of 178 N in the anterior region.

### 2.4. Methodology Proposed

The hypothesis employed for the development of the current study was the following:

1. Literature available about fatigue is experimental in most cases. Therefore, equations that describe material behaviour under cyclic loads cannot be too much realistic.
2. Fatigue studies in dental implants available in the literature are addressed from a deterministic point of view. Stochastic variations of the geometry and dimensions, material properties, and load history have a decisive influence on the fatigue phenomenon in dental implants, inducing important deviations from the mean or characteristic values of the fatigue life when considered as deterministic [29].
3. Fatigue is therefore recognised as a random process, which only recently has started to be analysed with the tools of the probability theory.

Steps to obtain the results with the methodology employed in the present study are the following:

- Obtain the mesh of the geometry by the used of ANSYS.
- Apply boundary conditions and loading configuration with ANSYS.
- Apply the probabilistic finite element method with the aim of obtaining the statistics of the stress.
- Apply the cumulative damage model to obtain the mean life, the variance, and the probability of failure.

The reader is referred to Prados-Privado et al. [19] for further details.

## 3. Results and Discussion

Probabilistic finite element method proposed in the current study has been applied on Proclinic® dental implants. Two different situations have been studied: fatigue behaviour in molar and fatigue behaviour in anterior region. Magnitude forces employed are shown in Table 4 and these loads were applied with three different angles. All results shown here have been measured in the neck (point A), body (point B), and apical region (point C).

### 3.1. Stress Distribution

**Figure 4:**
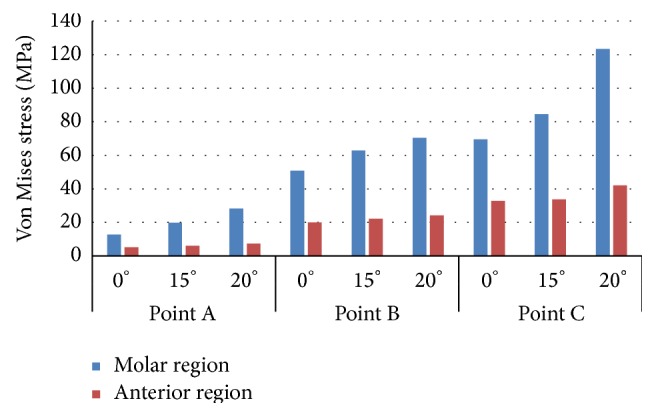
Von Mises stress in implant IP861.

**Figure 5:**
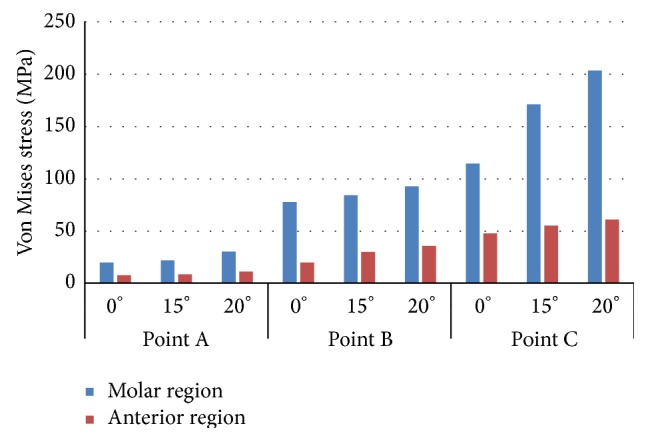
Von Mises stress in implant IP804.

Von Mises stresses on implants were used to assess the stress distribution in each situation. The Von Mises stress values in each point of study are shown Figures 4 and 5. When the stress distribution in both implants was compared, it was found that all investigated stress values in molar region were higher than the values in anterior region. Maximum Von Mises stress values were found in both dental implants and in each point when the static load is applied with the maximum angle.

### 3.2. Mean Fatigue Life and Variance

**Figure 6:**
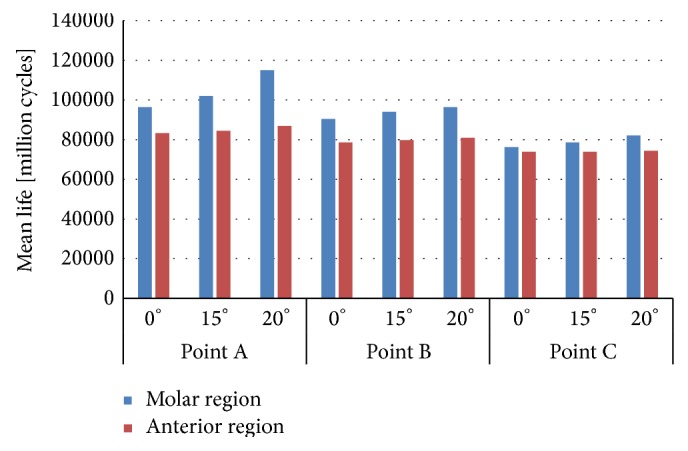
Mean fatigue life in implant IP861.

**Figure 7:**
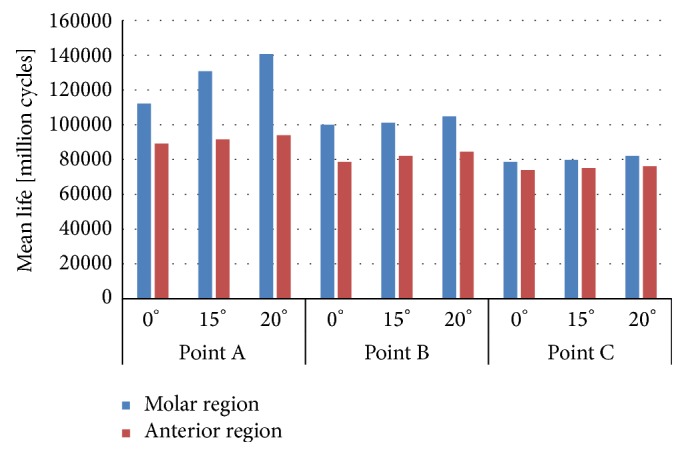
Mean fatigue life in implant IP804.

Applying the model proposed here, which was explained in detail in [19], the mean fatigue life and its variance have been obtained in all situations described. Figures 6 and 7 depict the mean fatigue life for dental implants employed. The most breakable part of the implant, independently of the configuration load, is the apical part. However, under the same loading conditions, the minimum mean life is relatively similar under the same situation in both dental implants.

**Figure 8:**
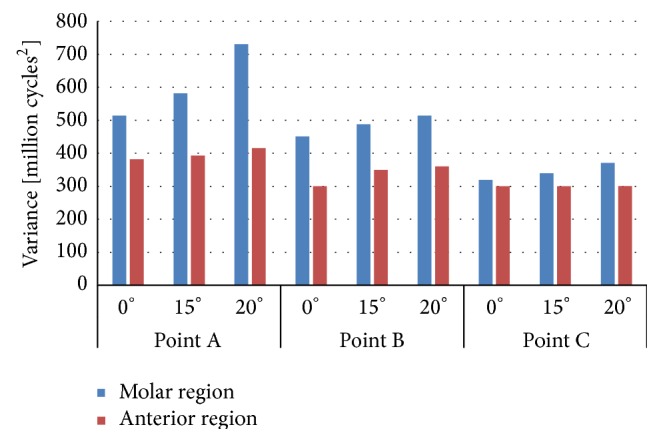
Variance of the mean fatigue life in implant IP861.

**Figure 9:**
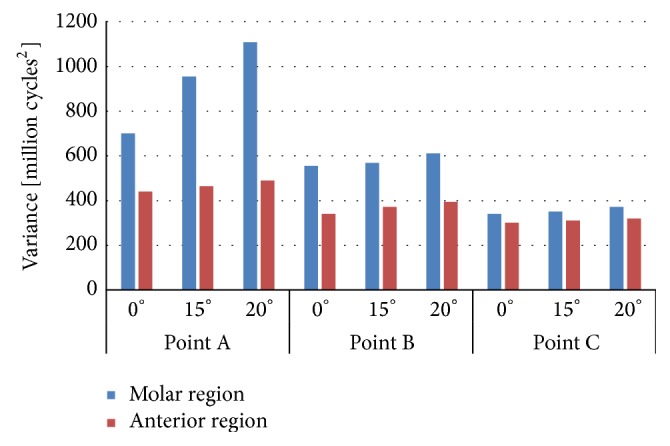
Variance of the mean fatigue life in implant IP804.

With the aim of having a good accuracy on the results, fatigue life must be correctly defined by statistic parameters. Therefore, the variance was also obtained and represented in Figures 8 and 9. Cylindrical implant (IP804) has more variability in terms of fatigue than the conical implants under the same situation of boundary conditions and loading configuration.

### 3.3. Probability of Failure

**Figure 10:**
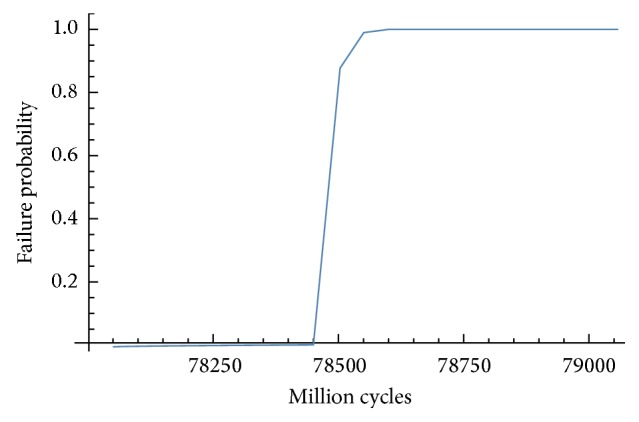
Cumulative probability function for implant IP861 and axial molar load.

**Figure 11:**
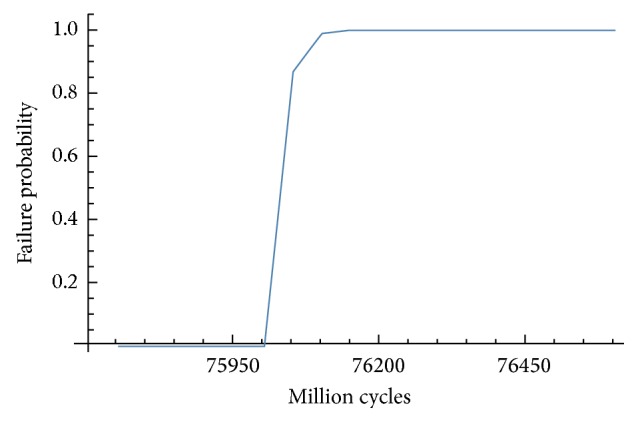
Cumulative probability function for implant IP804 and axial molar load.

Once the statistic parameters of the long-term life are defined, the probability cumulative function can be drawn. Two examples, which correspond to axial load situation, are shown in Figures 10 and 11.

In view of Figures 10 and 11, implant IP861 has a better behaviour in terms of failure because this implant has more cycles with a probability of failure equal to zero.

## 4. Discussion

The present study focused on the problem of the fatigue behaviour in different areas of the jaw. This paper presents the application of a probabilistic methodology to dental implants with the aim of knowing the fatigue behaviour and the probability of failure under two different loads with three different angles of application. The methodology employed offers a technique to define the influence of the variability and uncertainty of the most important factor in the efficacy of dental implants performance.

Different from most of studies available on the literature about fatigue in dental implants, this study has been proposed from a probabilistic point of view. As forces act on a repeated way, fatigue failure is introduced in dental implant [30]. Mastication habits are also different depending on the patient [22–28, 31]. Therefore, this decision is justified because dental implants have stochastic characteristics and, also, because they are very sensitive to many factors such as load and material. As opposed to the conventional way of studying fatigue, our results provided the mean fatigue life, its variance, and the probability of failure associated with each cycle.

Probabilistic models on dental applications are relatively new; thus, most of finite element and fatigue studies on dental implants available on the literature are deterministic [26, 32–34].

A realistic finite element model has been applied in the present work with the goal of obtaining the mean fatigue life, its variance, and the probability of failure. The influence of the material properties and the loading conditions on probability of failure has been demonstrated.

Several assumptions have been made with regard to the material properties and model generation. Implant material properties were assumed to be homogenous, linear, and isotropic and were assumed to be 100% osseointegrated.

Our results showed that the stress was mainly concentrated at the apical part of the implant when the highest angle was applied. The mathematical model proposed in the current work provided a relative similar mean life in all implants analysed under the same situation. However, the variance of the long-term fatigue life suggests that cylindrical implants have more variability because their variance is bigger than the values obtained in conical implants. According to the clinical experience, the conical implant employed here has a better behaviour than the cylindrical.

Finally, the cumulative probability functions were obtained. These functions provide the failure probability associated with each cycle in a determined condition. The model proposed in this study is focused on the study of fatigue with a probabilistic point of view obtaining accuracy results.

## 5. Conclusions

Variables involved in implant behaviour introduce randomness in the process due to the fact that masticatory forces are not constant and material properties can be different along the implant. Method to study fatigue employed here reduces the unrestrained elements.

To be able to quantify the randomness in this process, a cumulative damage model based on Markoff chains and the probabilistic finite element have been applied. This is the novelty of this paper because most finite element studies on dental implants are static analyses [34–38].

Proclinic® dental implant has been studied under two different load magnitudes, one bruxism and one a common masticatory load. As it was expected, stresses in all benchmark are bigger under bruxism condition. In light of the results of this study, the cylindrical implants have a greater uncertainty in the fatigue process, which is reflected in greater probability of failure.

## Figures and Tables

**Table 1:** Characteristics of each implant.

Implant name	Connection	Diameter
IP861	External hexagon	4.1
IP804		3.3

**Table 2:** Bite forces on molar region in the literature.

According to	Maximum bite force [N]	Range age [year]
[22]	300–600	10–70
[23]	387.79–392.34 (mean) 836–884 (maximum)	20–50 (mean = 24.89; SD = 5.658)
[24]	497.3–629.65	15–18
[25]	583,49	—

**Table 3:** Bite forces on anterior region in the literature.

According to	Maximum bite force [N]
[26]	150
[27]	146
[28]	178

**Table 4:** Bite force magnitude and angle employed in this study.

	Magnitude force [N]	Angle [°]
Molar region	489 [25]	0, 15, 20
Anterior region	178 (maximum value in the literature) [28]	0, 15, 20
